# Reconstruction of a Complex Posterior Tracheal Wall Defect via Transtracheal Running Suture and Pedicled Pectoralis Major Muscle Flap

**DOI:** 10.70352/scrj.cr.24-0009

**Published:** 2025-02-18

**Authors:** Tomoyuki Nakagiri, Alaa Selman, Tobias Goecke, Hayan Merhej, Akylbek Saipbaev, Arjang Ruhparwar, Patrick Zardo

**Affiliations:** 1 Department of Cardiothoracic, Transplantation, and Vascular Surgery, Hannover Medical School, Hannover, Germany; 2 Biomedical Research in Endstage and Obstructive Lung Disease Hannover (BREATH), Member of the German Center for Lung Research (DZL), Hannover, Germany

**Keywords:** tracheal reconstruction, tracheal posterior wall defect, complication of a tracheostomy, muscle flap, pectoralis major muscle

## Abstract

**INTRODUCTION:**

A tracheal membranous injury is a known complication of tracheostomy. After esophageal resection, such injury may prove fatal. No natural buttressing of the lesion occurs, and severe sepsis and mediastinitis may occur. In these situations, a circumferential tracheal resection is the treatment of choice, sometimes on cardiopulmonary bypass. However, the outcome is not always favorable.

**CASE PRESENTATION:**

We report a case of a long tracheal membranous wall defect (> 7cm) after esophageal resection. We successfully performed a transtracheal direct repair of the defect through a partial sternotomy, and reconstructed the ventrolateral wall with a muscle flap using the right pectoralis major muscle.

**CONCLUSION:**

Tracheal reconstruction through a T-shaped incision and anastomotic buttressing using a pectoralis major muscle flap could prove to be useful when reconstructing a posterior tracheal wall injury, especially after esophageal resection.

## Abbreviations

PDT
: percutaneous dilatational tracheostomy
ICS
: intercostal space
ST
: surgical tracheostomy

## INTRODUCTION

Tracheal injury is a possible complication of long-term intubation and tracheostomy.^1)^ It often relates to long-term tracheal wall contact of the intubation/tracheostomy-tube with unsuitable cuff pressure or at the insertion of the tube. When the patient is still under sedation, it may elude detection and lead to a tracheal defect. It usually arises at the posterior wall, as it has less tissue strength, compared to the tracheal cartilage, and the anatomical location. Normally, in this place, it is covered through the esophagus, which has good blood flow and provides natural buttressing.

After esophageal resection, the condition is different. The procedure itself is prone to complications, including anastomotic leakage and secondary bronchial dehiscence, which, in turn, may lead to long- term mechanical ventilation. In these circumstances, a post-tracheostomy posterior wall defect directly faces the mediastinal tissues or to the former esophageal cavity. In addition, the blood supply of the trachea from the esophagus is absent. Chronic inflammation additionally may enlarge the defect, which, in turn, leads to mediastinitis and sepsis.

We experienced a case of intrathoracic tracheal membranous wall defect after esophageal resection. In this situation, we performed a transcervical transtracheal direct repair of the defect and reconstructed the trachea with a pedicled muscle flap using the right pectoralis major muscle.

## CASE PRESENTATION

A 64-year-old male patient without any comorbidities suffering from a GIST (pT1, N0, Mx) and an incidentally discovered esophageal carcinoma (cT3, N1, Mx) had undergone thoracic esophageal resection, and gastric pull-up with Ivor–Lewis–Procedure,^2)^ with which the stomach tube is pulled through the hiatus into the posterior mediastinum where the esophagus was removed, and is anastomosed with the cervical esophagus in the chest, via abdominal robotic surgery and right thoracotomy. After an initially uneventful course, adjuvant chemotherapy with carboplatin and paclitaxel, and radiation therapy was administered. This led to a secondary bronchial fistula with the stomach, prompting us to perform a right main bronchus sleeve resection with gastrectomy and esophagostomy via right re-thoracotomy. After long-term sedation and tracheostomy with percutaneous dilatational tracheostomy (PDT), a long (>7 cm) tracheal membranous wall defect developed, and formed a purulent blind fistula to the former esophageal cavity. The patient was referred to our department for consultation.

**Fig. 1:**
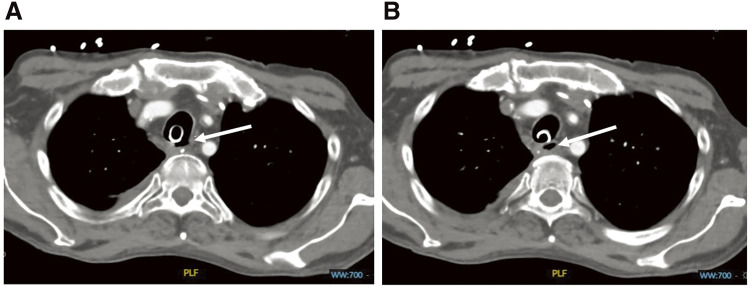
Tracheal posterior wall injury (**A**) and fistula (**B**). (**A**) Defect of the tracheal posterior wall (origin of the fistula). The length of the tear was approximately 7 cm. (**B**) The blind fistula was covered with a white matter bronchoscopically resembling an ulcer (no figure). It extended caudally about 2 cm.

Bronchoscopy and CT showed a long longitudinal defect in the posterior wall with a blind fistula (**Figs. 1A** and **1B**). We opted for direct identification of the lesion through a T-shaped transection of the ventral wall of the trachea, a transtracheal reconstruction, and buttressing of the anastomosis with a muscle flap of the right pectoralis major muscle.

**Fig. 2:**
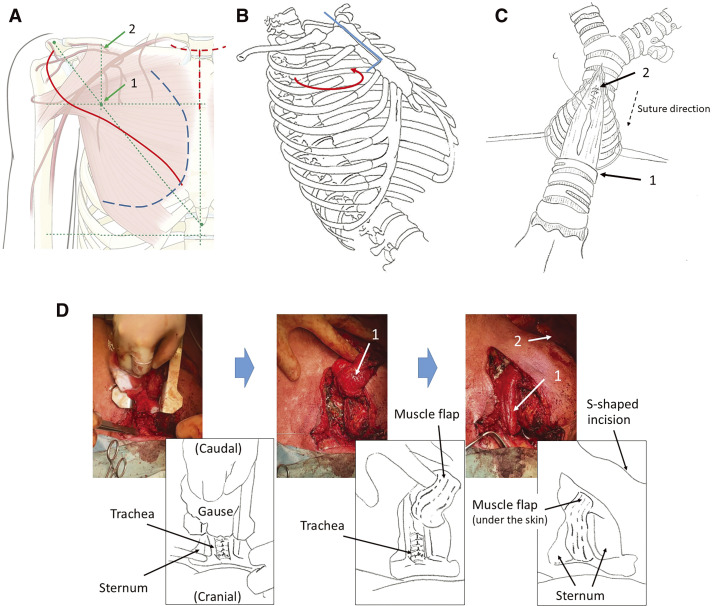
Surgical procedure. (**A**) Illustrations of the skin incision and the muscle flap. The acromion (green leftmost point) and xyphoid (green rightmost point) were marked. An S-shaped incision was made along the Ariyan-line (the line between both marks: green dotted line between them) from axillary to parasternal at the level of the nipple on the right side (red solid line). The point of inflow of the main blood vessel into the pectoralis major muscle, which is at the intersection point (1) between the line of the lateral 1/3 of the clavicle (2) and the oblique line (the green dotted oblique line), must be protected to keep a blood supply. In addition, a cervical incision and small median incision (Y-shaped) were made for the tracheal intervention (red dotted line). To harvest a muscle flap, the lateral edge of the pectoralis major muscle was exposed. It was dissected medially to expose the muscle and develop the lateral vascular pedicle. The dissection was extended in a cranial direction while protecting the pedicle (blue dotted line). (**B**) Partial sternotomy and partial chest wall resection. A partial upper sternotomy with partial transection to right side in the third ICS was made because the defect extended nearly to the main carina (blue line). The pectoralis major muscle flap was inserted through the ICS and was pivoted from retrosternal to the left paratracheal and pretracheal space (red curved arrow) via a partial medial chest wall resection. (**C**) The suture of the posterior wall defect. The trachea was opened with a T-shaped incision. The transverse incision of the trachea was made on the level of the defect ventrally (arrow 1). A single knot suture was made on the caudal edge of the tear (arrow 2). The posterior wall defect was sutured continuously from caudal end to cranial (dotted arrow). Evermann *et al*. propose to make a running suture from cranial to caudal.^6)^ However, we recommend our suture direction because of an easier handling. At the end of the suture, a single knot suture was made to avoid further tearing. In addition, we used 4/0 PDS to close the defect because we closed in single layer technique. For better maneuverability, 5/0 PDS could be also considered, even though the tensile strength may not suffice. (**D**) Pectoralis major muscle flap on the anastomosis (chronologically left to right; downward is cranial). The trachea was closed with single knot suture (left). Because the muscle was thick and broad (middle, arrow 1: pectoralis major), we performed a partial medial chest wall resection (third rib resection). The muscle flap covered the trachea (right, arrow 1: pectoralis major). The pectoralis minor and fat tissue could be seen through the S-shaped incision (arrow 2). ICS, intercostal space

In supine position with the head reclined, conventional orotracheal intubation was performed, and tube position bronchoscopically controlled. With the acromion and xyphoid as landmarks, an S-shaped incision was made along the Ariyan-line^3)^ from axillary to parasternal at the level of the nipple on the right side (**Fig. 2A**). The lateral edge of the pectoralis major muscle was exposed and dissected medially to expose the pectoralis minor muscle and develop the lateral vascular pedicle. The dissection was extended in a cranial direction while protecting the pedicle (**Fig. 2A**). A cervical T-shaped incision with careful dissection was made to expose the cervical trachea. Due to extensive secondary goiter, a right-sided hemithyroidectomy was performed. Because the defect extended nearly to the main carina, a partial upper sternotomy was performed with transection of the sternum at the level of the third intercostal space (ICS) (**Fig. 2B**). The front of the trachea was exposed with a punching defect still present at the level of the original tracheostomy. The transverse incision of the trachea was made on the level of the defect ventrally and a longitudinal incision carried out caudally (**Fig. 2C**). A spiral tube (6.0-mm internal diameter) was inserted, and cross-field-ventilation initiated. First, the posterior wall defect was sutured continuously from caudal to cranial end with a 4/0 PDS running suture, with single knot stitches at the caudal and cranial ends (**Fig. 2C**).

**Fig. 3:**
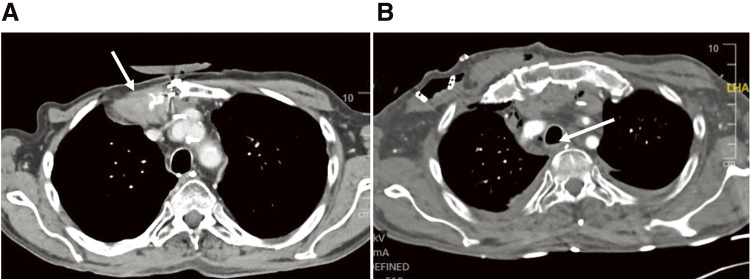
Postoperative CT. (**A**) The pectoralis major muscle was inserted in the thoracic cavity through the third ICS with a partial chest wall resection (arrow). (**B**) The posterior wall was competed and the fistula was completely removed (arrow). ICS, intercostal space

Reconstruction of the anterior wall was performed using 3/0 PDS in single knot technique. Prior to completing the anastomosis, we removed the cross-field tube and placed an orotracheal tube under direct visual and bronchoscopic observation with attention to place the cuff caudally of the suture. A water test was unremarkable and confirmed a patent anastomosis. After a wash with Polyhexanide solution (Serasept), the left paratracheal defect, located in the former esophageal bed, was filled with a fibrin glue/antibiotic mixture. The right pectoralis major muscle was pivoted from retrosternally into the left paratracheal space and fixed with the fibrin glue, and pretracheally via a partial medial chest wall resection (third rib; **Figs. 2B, 2D**, and **3A**) to buttress the anastomosis. The muscle was then fixed and the wound was closed in layers after drain placements.

At the end of the procedure, there was venous congestion and anisocoria, so that a neck/head and chest CT were carried out in the immediate postoperative course (**Figs. 3A** and **3B**). Shortly after the CT scan, all symptoms gradually disappeared.

At bronchoscopic follow-up 6 months after the procedure, no residual tracheal defect was present.

## DISCUSSION

Posterior tracheal wall injury during tracheostomy with an incidence of 12.5% in an animal study is a well-known complication.^4)^ However, a clinically apparent transmural tracheoesophageal fistula is very rare,^1)^ probably because most posterior lacerations are inherently buttressed by the esophagus. The esophagus itself benefits from a good blood supply from the inferior thyroid artery and the thyrocervical trunk of the subclavian artery to the cervical part and directly from the aorta toward the thoracic part. As most posterior wall injuries are small and do not affect the esophagus itself, they will heal without intervention.^1)^ However, large tears may be a life-threatening complication with airway bleeding, mediastinal emphysema and mediastinitis. In that case, surgical intervention is needed, and may even require cardiopulmonary bypass, which may be unsuitable for critically ill patients. Potential salvage procedures include placement of self-expanding metallic stents and/or Montgomery tubes.^5)^

In cases of prolonged orotracheal intubation and mechanical ventilation, tracheostomy is considered. Recently, the procedure is performed as PDT. The incidence of posterior tracheal injury is higher in PDT than conventional open surgical tracheostomy (ST).^1)^ As patients after esophagectomy and gastric pull-up have an impaired regional blood supply and a residual cavity in place of the former esophageal bed, the injury could worsen and additionally suffer from locoregional infections. PDT is generally considered to be a simpler procedure than ST and is widely accepted, especially in the intensive care unit setting. This, in turn, increases the chances of us encountering this sort complication with an extensive posterior tear.

For the reconstruction, a right thoracotomy could have been considered; however, several factors made this approach less favorable. First, the defect was located slightly on the left side, and repairing the tear via a right thoracotomy would be challenging due to limitations in the surgical field. Second, there was a risk of exacerbating the defect by inserting a bronchial tube for one-lung ventilation. Third, the defect could be infected, and the surrounding tissue was fragile, necessary careful avoidance of tension on the left membranous part during suturing. Specifically, suturing the membranous part of the trachea via a right thoracotomy might require manipulating and pulling the left membrane to align it with the right side for suturing. In addition, adhesions in the right thoracic cavity, particularly around the right main bronchus, were anticipated due to the patient’s history of previous thoracotomies and sleeve resection. These adhesions could restrict the mobilization of the lung and trachea. For these reasons, this anterior approach was ultimately selected.

Evermann *et al*. reported a special technique with a T-shaped incision on the ventral tracheal wall to suture a longitudinal membranous wall injury.^6)^ The operation can be indicated also for an injury more than 3 cm and can be performed without cardiopulmonary bypass or extracorporeal membrane oxygenation.

To effectively buttress an intrathoracic anastomosis and/or a bronchial stump, an intercostal muscle flap, the latissimus dorsi, serratus anterior muscle, or pericardial tissue (fat and/or pericardium) have been described.^7)^ However, in this specific case, neither of these approaches were suitable to covering the anastomosis and/or the suture. Due to gastric pull-up, omentum could not be harvested, and the right latissimus dorsi muscle was transected during the initial Ivor-Lewis-Procedure. This led us to buttress our bronchial sleeve with a modified diaphragmatic muscle flap, thus effectively limiting our options. Pectoralis major muscle flap is useful in head and neck reconstructive procedures, by otolaryngology Surgeons. But, also, thoracic surgeons may rely on this specific flap for intrathoracic procedures.^5)^

Our patient had already undergone several operations, and was additionally impaired by local infection. Neither a stent implantation nor cardiopulmonary bypass was a suitable approach. We opted for the previously described technique with a T-shaped incision of the ventral tracheal wall and a buttressing with a pedicled pectoralis major flap.

## CONCLUSION

Tracheal reconstruction through a T-shaped incision and anastomotic buttressing using a pectoralis major muscle flap could prove to be useful when reconstructing a posterior tracheal wall injury, especially after esophageal resection.

## DECLARATIONS

### Funding

Not applicable.

### Authors’ contributions

TN: writing—original draft, data curation, methodology, and investigation.

AS: data curation.

TG: data curation.

HM: data curation.

AS: data curation.

AR: data curation and supervision.

PZ: conceptualization, supervision, and writing—review and editing.

All authors have read this manuscript and have approved this submission.

### Availability of data and materials

Not applicable.

### Ethics approval and consent to participate

This work does not require ethical considerations or approval. In addition, the patient has permitted us to publish this case report.

### Consent for publication

The patient has permitted us to publish this case report.

### Competing interests

No conflict of interests.
